# The case for compensatory processes in the relationship between anxiety and error monitoring: a reply to Proudfit, Inzlicht, and Mennin

**DOI:** 10.3389/fnhum.2014.00064

**Published:** 2014-02-28

**Authors:** Jason S. Moser, Tim P. Moran, Hans S. Schroder, M. Brent Donnellan, Nick Yeung

**Affiliations:** ^1^Department of Psychology, Michigan State UniversityEast Lansing, MI, USA; ^2^Department of Psychology, University of OxfordOxford, UK

**Keywords:** anxiety, worry, error-related negativity, error monitoring, cognitive control, emotion, motivation

We thank Proudfit et al. (2013) for their thoughtful response to our Review. The gist of their commentary is that motivation and emotion play a larger role in the relationship between anxiety and the error-related negativity (ERN) than conceived in our compensatory error-monitoring hypothesis (CEMH; Moser et al., 2013). Here we respond to Proudfit et al.'s commentary in three ways:

1. We clarify that our view does in fact place motivation and emotion center-stage in the anxiety-ERN relationship.
2. We reiterate that there is currently little compelling evidence that an enlarged ERN in anxious individuals reflects “the disposition to respond more strongly to uncertain threat” (Proudfit et al., 2013, p. 1).
3. We emphasize that our analysis focuses specifically on the functional significance of the elevated ERN characteristic of many anxious individuals.

As in our original review (Moser et al., 2013), we treat anxiety as a multi-faceted dimensional construct that varies from mild to severe, with anxiety disorder patients falling at the severe end of this continuum (Watson, 2005; Brown and Barlow, 2009). The foundation of our CEMH is that an enlarged ERN is not related to all forms of anxiety, instead it is linked primarily to the anxious apprehension or worry facet of anxiety (Nitschke et al., 2001; Moser et al., 2012). Indeed, our meta-analysis indicated that worry, obsessions, and related future-oriented catastrophic thoughts showed the strongest association with enlarged ERN. Furthermore, we believe that motivation and emotion play an important role in the relation between this facet of anxiety and the ERN. Specifically, we suggest that worry, a negatively valenced cognitive-emotional construct (Newman et al., 2013), simultaneously depletes working memory and enhances compensatory processes aimed at counteracting this depletion (cf. Eysenck et al., 2007; Vytal et al., 2013). Our view is that anxiety-related modulation of the ERN results from this compensatory effort. Thus, our CEMH considers emotion and motivation as important players in the anxiety-ERN relationship, counter to Proudfit et al.'s characterization of our view.

We also provided a range of evidence supporting the CEMH. In particular, we showed (1) that worry-related anxiety was associated with an enlarged ERN in the presence of unaltered behavioral performance, (2) that loading working memory with non-valenced information (a neutral analog for the effects of worry) also enlarged the ERN, and (3) that worriers with an enlarged ERN performed better in school than worriers with relatively smaller ERNs, but similarly to non-worriers. These results strongly suggest that an enlarged ERN among individuals high in worry serves a compensatory function.

The findings in support of our CEMH are not well explained by Proudfit et al.'s perspective that an enlarged ERN in anxiety reflects a heightened threat response. Principally, their perspective does not account for the meta-analytic finding that worry demonstrates the most robust link with the enlarged ERN. In fact, their view is that worry is not necessary for enhanced ERN in anxiety. It is likewise unclear how their position would explain the other findings we reported in support of our CEMH (listed above). Thus, our first concern with Proudfit et al.'s conceptualization is that their threat sensitivity proposal does not capture a number of anxiety-ERN findings.

Our second reservation with Proudfit et al.'s perspective is that existing research does not clearly support their view that an enlarged ERN in anxiety reflects a heightened threat response to errors. To make the case that modulation of the ERN reflects enhanced sensitivity to errors in anxiety there needs to be convincing evidence that anxiety, ERN, and other (potentially intervening) measures of threat sensitivity are interrelated. To date, little compelling evidence exists for this multivariate relationship. Specifically, the ERN is uncorrelated with other physiological markers of defensive responding to errors, including error-related skin conductance, heart rate changes (Hajcak et al., 2003), or error-potentiated startle (Riesel et al., 2013). In fact, we reanalyzed the significant ERN-startle relationship reported by Hajcak and Foti (2008) and show that the observed relationship was dependent on a single outlier (*r* = −0.07 vs. *r* = −0.38 with the outlier included; See Appendix for details of this reanalysis). The ERN is also unrelated to perfectionistic concerns about mistakes (Pieters et al., 2007; Schrijvers et al., 2010; Tops et al., 2013), the tendency to view personal mistakes negatively and to interpret them as failures (Frost et al., 1990). This is important because perfectionistic concerns about mistakes is an individual difference that should have direct relevance to the ERN if the ERN is conceptualized as a disposition to react more negatively to errors. Although many forms of anxiety do correlate with concerns about mistakes (Antony et al., 1998), just because anxiety is related to self-reported concerns about mistakes does not mean enlarged ERN reflects this perfectionistic concern in anxious individuals. Moreover, concerns about mistakes seems to cut across a number of different anxiety problems whereas our meta-analysis suggests increased ERN is more specific to worry-related anxiety.

To provide evidence for their threat sensitivity hypothesis Proudfit et al. cite a study wherein undergraduates scoring high on negative affect showed increased ERN and increased error-related skin conductance (Hajcak et al., 2004). However, there are potential limitations with this conclusion. First, the relationship between negative affect and the ERN is unclear—Luu et al. (2000) actually found that negative affect was associated with an overall smaller ERN. Second, although individuals high in negative affect showed increased ERN and error-related skin conductance what was missing was a correlation between ERN and error-related skin conductance in the high negative affect individuals, which could have provided more direct support for the multivariate relationship Proudfit et al. suggest.

Perhaps most problematic for their view are the data showing that obsessive compulsive patients fail to demonstrate an especially large ERN to errors that are punished with monetary loss (Endrass et al., 2010). Specifically, Endrass et al. showed that whereas non-anxious control participants evidence an enlarged ERN during a condition in which errors were punished with monetary loss compared to a no punishment condition, obsessive compulsives failed to evidence any change in ERN between conditions. Why is this null result so important? Because, if the ERN is modulated by what Proudfit et al. refer to as “affect that is integrally related to errors” (p. 2), which includes when errors are punished, and anxious individuals are characterized by increased threat response to errors, then it follows that the obsessive compulsives should have demonstrated a larger ERN to punishment compared to a no punishment condition more so than controls. However, this was not the case. In fact, the null finding is directly supportive of our CEMH and consistent with Eysenck et al.'s attentional control theory (Eysenck et al., 2007) on which our CEMH was largely based. That is, anxious individuals are proposed to engage in compensatory effort during standard conditions—resulting in an enlarged ERN—and thus cannot exert additional effort in a more effortful punishment condition—because they are already at ceiling—the net effect of which would be no ERN modulation across conditions.

Flipping this “integral affect” notion on its head, Proudfit et al. cite Bartholow et al. (2012) who showed that alcohol ingestion reduced the magnitude of the ERN via its reduction of negative affect. Proudfit et al. argue that these findings suggest that “when [participants] ingest an anxiolytic agent that leads them to care less about their errors, the ERN decreases” p. 2. Although Bartholow et al.'s findings are suggestive that ERN is sensitive to changes in state negative affect, we take issue with the conclusion that the reduction in negative affect resulting from ingesting alcohol reduced ERN because of reduced concern about mistakes. The general reduction of negative affect in the alcohol group does not necessarily imply that participants cared less about their mistakes or that they experienced less affect related to errors, *per se*. For example, behavioral indicators from Bartholow et al. suggest that individuals in the alcohol group were well aware of their mistakes and even made appropriate adjustments to their mistakes compared to the no-substance control group. It also seems important to point out that, in fact, Bartholow et al.'s results may be supportive of our CEMH in that alcohol may have reduced worry and therefore reduced the ERN. The same may also be true of other studies showing that anxiolytic agents reduce the amplitude of the ERN (e.g., Johannes et al., 2001). Thus, although extant research suggests that ingestion of anxiolytic agents reduces the amplitude of the ERN, the mediating psychological mechanism is unclear at present and could certainly include reduction of worry. Regardless, our main point is that none of these findings directly speak to the role of this integral affect in the relationship between anxiety and enlarged ERN. The only data we are aware of that are relevant to the role of integral affect in the relationship between anxiety and enlarged ERN are those of Endrass et al. (2010) mentioned above, and their findings are not supportive of Proudfit et al.'s hypothesis, but rather fit with our CEMH.

Finally, the interesting developmental considerations outlined by Proudfit et al. do not rule out the possibility that enhanced ERN in anxiety reflects compensatory processes. Proudfit et al. have shown that the ERN is unchanged (Meyer et al., 2012), diminished (Torpey et al., 2013), or enhanced (Meyer et al., 2013) in anxious or anxiety-prone children under the age of 10, whereas it is clear that enhanced ERN characterizes anxious adults (Moser et al., 2013). Thus, it is unclear how the change in the anxiety-ERN relationship across development excludes the possibility that compensatory mechanisms link the two in adults. It is possible, for instance, that anxious children are characterized by diminished ERN, consistent with their tendency toward poor effortful control (e.g., Eisenberg et al., 2009), but later compensate for such impairment with enhancement of similar mechanisms to maintain some level of functioning (see also Gee et al., 2013 for a description of a similar compensatory developmental process). This is an interesting possibility we are currently examining in our laboratory.

We emphasize that developmental considerations are important for both theoretical and practical reasons. Likewise, we agree with Proudfit et al. that intensive longitudinal studies across significant periods of development will help clarify whether the ERN is a cause or a consequence of anxiety. On this point, if the ERN emerges as an endophenotype for anxiety it could certainly reflect compensatory processes. Nonetheless, we simply believe the existing developmental literature is too sparse and inconsistent to serve as a strong basis for drawing inferences about the functional relationship between anxiety and ERN.

To reiterate, the motivation in developing the CEMH was to understand the functional significance of the elevated ERN characteristic of many individuals with heightened anxiety. We do not attempt to provide an overarching account of the much broader and thornier relation between anxiety and threat. This is a critical distinction between our view and that of Proudfit et al. Our central claim, one that we believe is better supported by the existing data, is that the enlarged ERN characteristic of some anxious individuals and patients reflects compensatory cognitive control.

Finally, although we have outlined our disagreements with Proudfit et al.'s position, there is clearly benefit for this line of research—and for science in general—to having opposing views on the same findings. Future work in this area ideally should attempt to directly adjudicate between our view and that of Proudfit et al., as well as develop alternatives that may ultimately prove more accurate. Our view and that of Proudfit et al. also leverage somewhat different literatures—cognitive science/neuroscience versus affective-motivational science/ neuroscience, respectively—and so clearly a union of such synergistic perspectives will eventually be needed. At the heart of the anxiety-ERN relationship is likely an interplay of cognition and emotion/motivation, to the extent that the two are meaningfully separable (Shackman et al., 2011; Pessoa, 2013). Alternatively, it seems fair to say that motivated cognition is at the heart of the anxiety-ERN relationship.

## Figures and Tables

**Figure A1:**
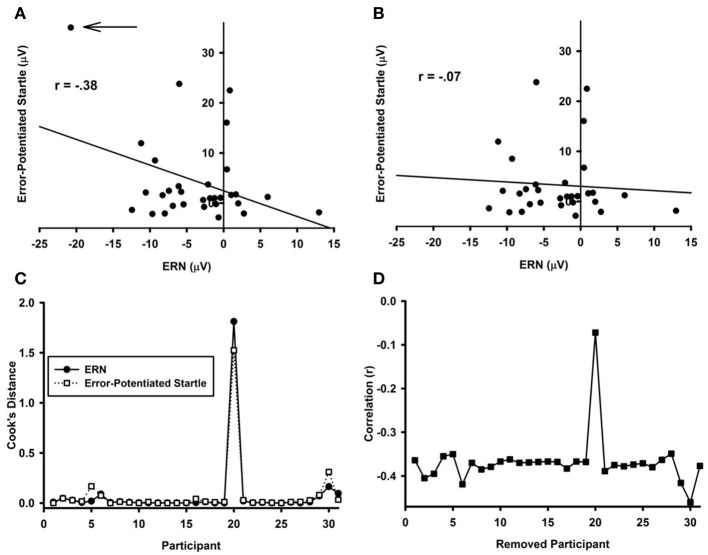
**(A)** The reproduced scatterplot; the arrow indicates an outlying data-point. **(B)** The reproduced scatterplot after the outlying data-point has been removed. **(C)** Cook's Distance for each participant. **(D)** The correlation between the ERN and error-potentiated startle calculated for the remaining 30 participants after each participant is iteratively removed.

## Appendix

Based on a visual inspection of the scatterplot in Figure 2 of Hajcak and Foti (2008), we identified a possible outlier that may have inflated the relationship between the ERN and error-potentiated startle. To confirm this impression, we reproduced the scatterplot using Dagra software (Blue Leaf Software, Inc.; see Altmann and Schunn, 2012 for an identical procedure) and assigned each participant an arbitrary ID number. The results of this procedure can be seen in Figure A1A.

We first computed Cook's Distance for each ERN and startle value. Cook's Distance measures the effect of deleting individual observations from a data set and is useful in identifying outlying and unduly influential data points (Cook and Weisberg, 1982). Guidelines on the interpretation of this statistic vary, but a value exceeding 1 is often used to identify outliers (Cook and Weisberg, 1982). As can be seen in Figure A1C, one participant (*ID* = 20) met this criteria for both the ERN (*D* = 1.8) and startle (*D* = 1.5) thereby corroborating the impression gleaned by visual inspection; this participant is identified in Figure A1A by the arrow. Finally, we iteratively removed individual participants and computed correlations between the ERN and error-potentiated startle for the remaining 30 participants. These data are presented in Figure A1D. Removing the outlier identified in the previous analysis reduced the −0.38 correlation to −0.07; when any other participant was removed, the correlation ranged from −0.34 to −0.46. The effect of removing this participant can be seen in Figure A1B.

Overall, the results of this reanalysis suggest that the association between the ERN and error-potentiated startle reported by Hajcak and Foti (2008) was highly dependent on a single data point. A conservative but reasonable interpretation is that there is little compelling evidence for an association between the ERN and error-potentiated startle in the Hajcak and Foti (2008) data.
